# Charomers—Interleukin-6 Receptor Specific Aptamers for Cellular Internalization and Targeted Drug Delivery

**DOI:** 10.3390/ijms18122641

**Published:** 2017-12-06

**Authors:** Ulrich Hahn

**Affiliations:** Chemistry Department, Institute for Biochemistry and Molecular Biology, MIN-Faculty, Universität Hamburg, Martin-Luther-King-Platz 6, D-20146 Hamburg, Germany; uli.hahn@uni-hamburg.de; Tel.: +49-177-213-4297

**Keywords:** aptamers, charomers, targeted drug delivery, targeted chemotherapy, photodynamic therapy, interleukin-6 receptor

## Abstract

Interleukin-6 (IL-6) is a key player in inflammation and the main factor for the induction of acute phase protein biosynthesis. Further to its central role in many aspects of the immune system, IL-6 regulates a variety of homeostatic processes. To interfere with IL-6 dependent diseases, such as various autoimmune diseases or certain cancers like multiple myeloma or hepatocellular carcinoma associated with chronic inflammation, it might be a sensible strategy to target human IL-6 receptor (hIL-6R) presenting cells with aptamers. We therefore have selected and characterized different DNA and RNA aptamers specifically binding IL-6R. These IL-6R aptamers, however, do not interfere with the IL-6 signaling pathway but are internalized with the receptor and thus can serve as vehicles for the delivery of different cargo molecules like therapeutics. We succeeded in the construction of a chlorin e6 derivatized aptamer to be delivered for targeted photodynamic therapy (PDT). Furthermore, we were able to synthesize an aptamer intrinsically comprising the cytostatic 5-Fluoro-2′-deoxy-uridine for targeted chemotherapy. The α6β4 integrin specific DNA aptamer IDA, also selected in our laboratory is internalized, too. All these aptamers can serve as vehicles for targeted drug delivery into cells. We call them charomers—in memory of Charon, the ferryman in Greek mythology, who ferried the deceased into the underworld.

## 1. Introduction

The multifunctional cytokine interleukin-6 (IL-6) consists of 183 amino acids and is in case of e.g., a skin lesion secreted by violated cells to signal this violation to recipient cells, thus inducing an inflammation followed by the healing process. The IL-6 signal is recognized by a highly specific IL-6 receptor (IL-6R) which is presented at the surfaces of certain cells. At least two further molecules of the nearly ubiquitously occurring glycosylated transmembrane protein gp130 are needed to result in the active complex for initiating signal transduction from outside the cell, finally into the nucleus to regulate corresponding gene expression (for review see [1,2,3]). One prerequisite of many receptors is their ability to exhibit a mechanism for desensitizing. IL-6R achieves this by internalization.

IL-6 mediated signal transduction is involved in many disease processes and is thus of high medical relevance. In some cases, one might wish to have a tool at hand to interrupt this signaling pathway. Candidates therefore are antibodies or even better, aptamers. Highlighting advantages and disadvantages of aptamers can be omitted in a special issue on aptamers and thus we can step directly into the projects which should be described here.

Our original plan, initiated by Stefan Rose-John, was to select aptamers specific for IL-6R aiming at getting a tool at hand to block IL-6 mediated signal transduction. Attempts to select aptamers with high specificity for IL-6R were successful for canonical and modified RNA (dissociation constants from 20 nM to 55 nM [4,5,6]) as well as for DNA aptamers (dissociation constant 490 nM [7]).

All these aptamers, however, did not inhibit IL-6 signaling at all but most RNA aptamers were internalized and thus could function as vehicles for cargo delivery into target cells.

Another kind of cell surface proteins chosen as targets for the selection of aptamers in our laboratory was α6β4 integrin. This is presented by epithelial cells, Schwann cells, keratinocytes and endothelial cells [8,9]. The α6β4 integrin can bind to laminin, which leads to the assembly of hemidesmosomes followed by stable adhesion via connecting the intracellular keratin cytoskeleton to the basement membrane [10,11]. The selected α6β4 integrin specific aptamer IDA was also internalized.

In addition to the aptamers discussed so far, a number of others have been selected and characterized that can also be used to shuttle a variety of drugs, liposomes and (nano) particles into cells. Among those are aptamers targeting prostate-specific membrane antigen (PSMA) [12] which served for the directed delivery of an appropriate siRNA where it was connected to [13]. Aptamers specific for mucin-1 [14], nucleolin [15] transferrin receptor [16] or αvβ integrin [17]—just to list some as representatives—served as vehicles for different kinds of drug delivery approaches.

We have recently presented an overview on aptamers to be used as drug delivery vehicles [18,19]; readers are also referred to excellent reviews of the systemic administration of aptamer-based therapeutics by Burnett and Rossi [20] and Catuogno et al. [21], Sun et al. [22], Gilboa et al. [23], Jiang et al. [24] and not least, recently by Kruspe and Giangrande [25,26].

For all those internalized aptamers exhibiting the capability for cargo delivery I here would like to introduce the term “charomers”.

In this brief review, however, solely aptamers selected in our laboratory and suitable as charomers will be dealt with in the following.

## 2. Interleukin-6-Recetor (IL-6R) Specific Aptamers

### 2.1. G-Quadruplex Forming Interleukin-6 Receptor (IL-6R) Specific Dimeric RNA Aptamers of 19 or 34 Nucleotides

#### 2.1.1. AIR-3A—An Aptamer Specific for IL-6R and Consisting of RNA

**Figure 1:**
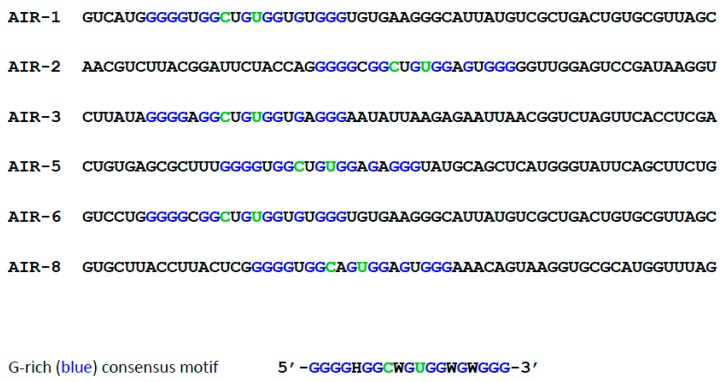
Alignment of interleukin-6 receptor (IL-6R) aptamer sequences from enriched pools. Consensus sequence is given below (conserved Gs in **blue** and conserved Cs and Us in **green**); H encodes A, C, or U and W encodes A or U, respectively. Flanking primer binding sites or constant regions of starting pool are omitted.

The first IL-6R specific aptamers selected in our laboratory consisted of RNA. Sequencing of the enriched pool revealed six individual clones all comprising a very similar consensus sequence (Figure 1; [4]).

**Figure 2:**
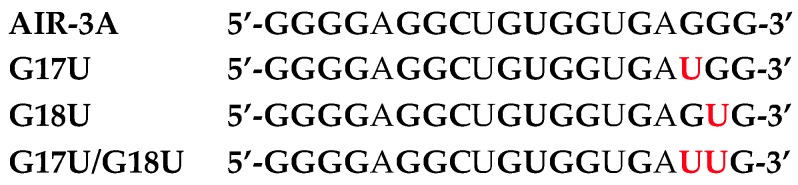
Nucleotide sequence of AIR-3A, the minimized active version of the IL-6R specific RNA aptamer AIR-3 and inactive AIR-3A variants; replaced nucleotides in red. A dissociation constant of about 20 nM was determined if AIR-3A was incubated with recombinant soluble human receptor (shIL-6R) in filter retention assays [4]. If the aptamer was incubated with IL-6R-presenting bone marrow-derived pro-B (BaF3) cells, the *K*_d_ turned out to be about 2 nM [27]. Variants with one (G17U or G18U) or two Gs replaced by Us (G17U/G18U), respectively, did not bind to any target at all.

**Figure 3:**
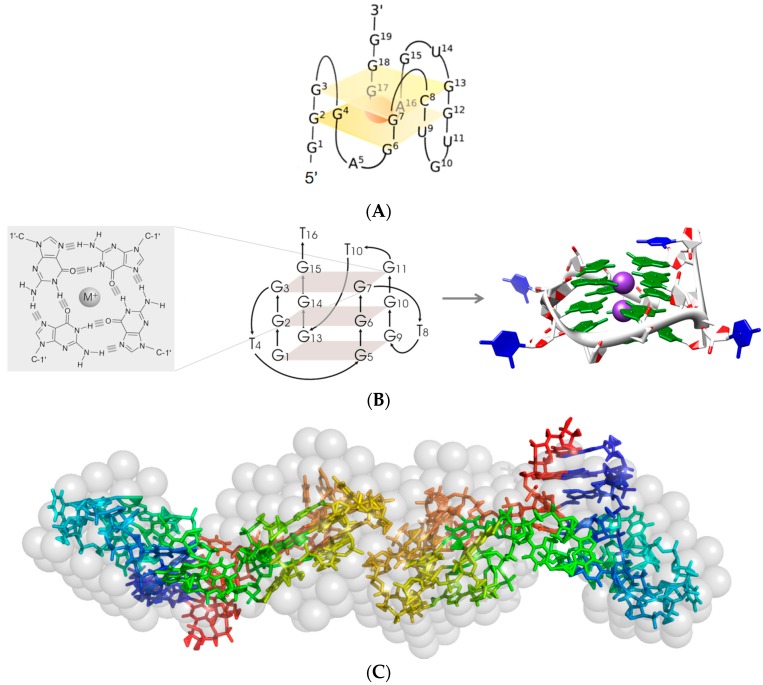
Aptamers AIR-3A, AID-1 as well as RAID3 all exhibit a G-quadruplex structure; the RNA aptamers AIR-3A and RAID3 were shown to dimerize. Circular dichroism (CD) spectroscopic investigations and UV-melting analyses revealed a G-quadruplex structure for both the RNA aptamers AIR-3A (**A**) [4] and RAID3 (**C**) [6], as well as for the DNA aptamer AID-1 (**B**) [7]. Balls in B represent structure stabilizing metal ions; gray semitransparent spheres in C symbolize a model deduced from synchrotron-based small-angle X-ray scattering (SAXS) analyses which could be superimposed with an ab initio model of an aptamer dimer.

Minimal variants of each of these clones presenting each individual consensus motif were synthesized and analyzed for their capacity to bind IL-6R. AIR-3A (an aptamer specific for IL-6R and consisting of RNA; Figure 2) turned out to be the best candidate and was thus used for all further investigations [4]. Its high G-content was a strong hint of a G-quadruplex topology of this aptamer. Biophysical analyses like circular dichroism spectroscopy (CD) and UV-melting studies proved that AIR-3A adopted a parallel G-quadruplex structure (Figure 3).

#### 2.1.2. RAID3—An RNA Aptamer for Interleukin-6 receptor Domain 3

Another IL-6R specific 34 nt long RNA aptamer selected in our laboratory was RAID3 (RNA Aptamer for Interleukin-6 receptor Domain 3) [6]. It also exhibited a G-quadruplex structure and, most remarkably, could post-selectively be modified by replacing all pyrimidines by their 2′-fluoro analogs, resulting in the aptamer RAID3 2′-F-Py. Both mentioned aptamers did not show significant differences in their target binding ability (*K*_d_ about 50 nM both). RAID3 2′-F-Py, however, exhibited an exceptional stability over a period of two days in Dulbecco’s modified Eagle’s medium supplemented with 10% fetal bovine serum (DMEM 10% FBS) at 37 °C. Not to forget that even the unmodified aptamer, RAID3, had a relatively long half-life of up to five minutes under the same conditions [6]. 

### 2.2. AIR-3A and RAID3 Are Internalized by IL-6R Presenting Cells and thus Charomers Allowing Their Usage as Vehicles for Targeted Drug Delivery

**Figure 4:**
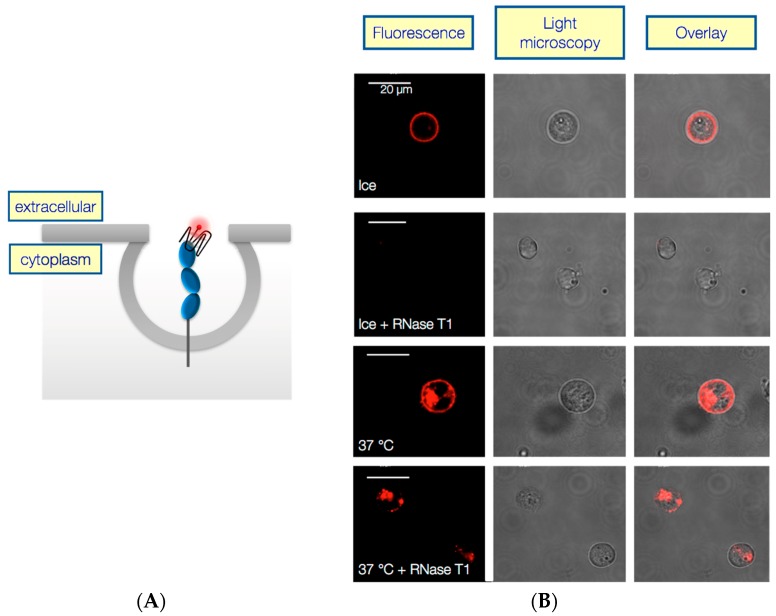
Fluorescently labeled aptamer AIR-3A is internalized by IL-6R presenting BAF/gp130/IL6R/TNF cells. (**A**) Schematic presentation of internalization process of G-quadruplex forming fluorescently labeled aptamer bound to the receptor IL-6R and (**B**) confocal laser scanning and light microscopy of IL-6R presenting cells after 30 min incubation with Atto645N-labeled AIR-3A at 37 °C and on ice (control, as internalization does not occur at 0 °C). Another control included an incubation with G specific ribonuclease (RNase) T1 which degraded surface bound RNA aptamers [4].

AIR-3A and also RAID3 both turned out not to interfere with IL-6 initiated signal transduction. IL-6R, however, was internalized [28] as are many other receptors or cell surface proteins. Therefore, it was obvious to assume that a considerably tight binding ligand might be internalized too, together with the receptor. This could be demonstrated for some of the IL-6R specific RNA aptamers selected in our laboratory (Figure 4 and [4,6]).

In Greek mythology, a ferryman named Charon ferried the dead from the world to the underworld. In memory of this ferryman and in honor of one of the first cloning vectors based on the bacteriophage lambda—which was invented by Blattner et al. in 1977 and named “Charon phages” [29]—and in search of an acronym, we named our internalized and drug delivering aptamers “charomers”.

### 2.3. Charomer Mediated Targeted Photodynamic Therapy (PDT)

**Figure 5:**
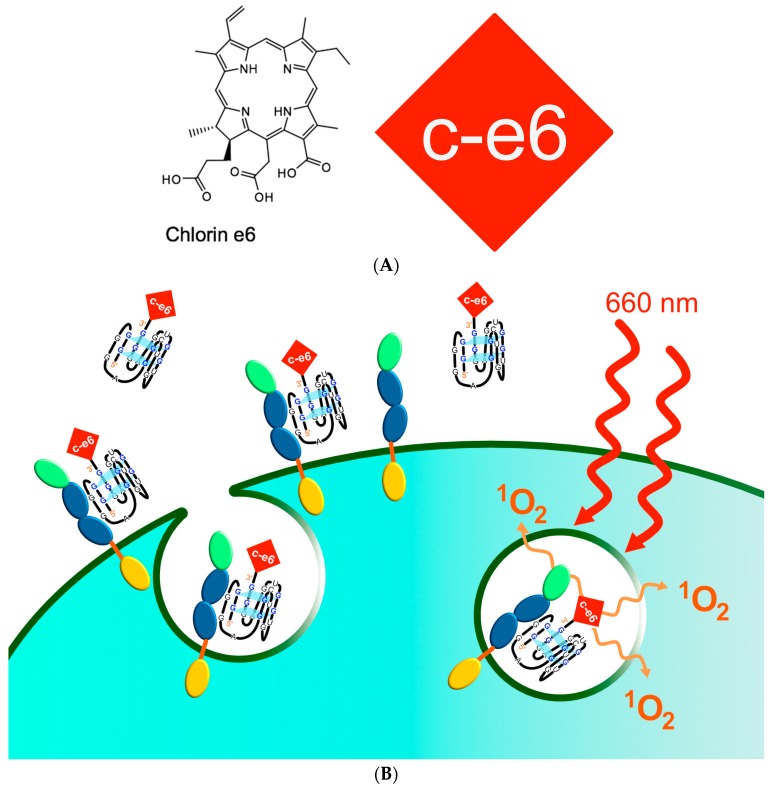
Chlorin e6 (c-e6) derivatized charomer AIR-3A-ce6 was internalized by IL-6R presenting cells leading to their destruction after illumination with appropriate light [34]. Schematic drawing of the aptamer mediated targeted photodynamic therapy (PDT). C-e6 (**A**) was covalently linked to the IL-6R specific aptamer (here schematically depicted as a G-quadruplex structured molecule); (**B**) This aptamer derivative was incubated with appropriate cells which did not survive after illumination with light of 660 nm wavelength (**red** waved lines) which is absorbed by ce-6 leading to the generation of singlet oxygen (^1^O_2_, **orange** waved lines) [34]; different colored ellipses of receptors symbolize different domains, extracellular domains in blue and green, intracellular part yellow.

Chlorin e6 (c-e6) is a photoactivatable agent that generates singlet oxygen upon irradiation (Figure 5A). It is approved for ex vivo and in vivo application and thus very well suited for photodynamic therapy (PDT [30,31,32,33]). If pure c-e6 is applied to target cells it is non-specifically internalized and intracellularly accumulated. We have covalently linked c-e6 to the 3′-terminus of the IL-6R specific RNA aptamer AIR-3A which was then incubated with IL-6R presenting cells for appropriate times. After illumination of treated cells with light of 660 nm cell vitality dropped considerably under 50% and apoptosis increased significantly [34].

### 2.4. Charomer Mediated Targeted Chemotherapy

**Figure 6:**
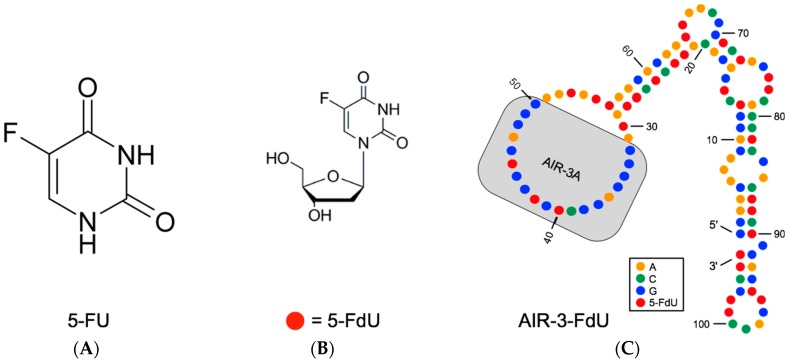
Charomer AIR-3-FdU, a tool for directed cancer drug delivery [27]. Shown are structures of 5-fluorouracil (5-FU; (**A**)), 5-fluoro-2′-deoxyuridine (5-FdU; (**B**)) and AIR-3-FdU (**C**) a derivative of the IL-6R specific RNA aptamer AIR-3 with each U replaced by 5-FdU (red dots); AIR-3-FdU structure is deduced from a model predicted for the originally selected aptamer AIR-3A with the software Mfold [39]; grey area emphasizes the minimized aptamer version AIR-3A.

The base analogue 5-fluorouracil (5-FU; Figure 6A) is a warts therapeutic [35] and known since 60 years as chemo therapeutic or cancer drug [36,37]. It is also used in different kinds of application forms [38]. We have enzymatically incorporated 5-fluoro-2′-deoxyuridine (5-FdU; Figure 6B) into aptamer AIR-3, the initially selected IL-6R specific “long version” of AIR-3A (Figure 1 and Figure 2). AIR-3 was chosen as it exhibits significantly more Us than AIR-3A (Figure 1 and Figure 6C). The resulting aptamer, AIR-3-FdU, still bound IL-6R with a dissociation constant of about 150 nM and IL-6R presenting BaF3 hIL-6R cells with a remarkable *K*_d_ of about 20 nM [27]. Furthermore, when incubated with target cells AIR-3-FdU also was internalized, finally resulting in a decrease of cell proliferation to about 75%.

To re-emphasize it, AIR-3-FdU could be readily synthesized in an enzymatic one step reaction. It specifically bound to a cell surface receptor which then most likely was transferred to the lysosome. When the aptamer then was degraded by intracellular nucleases, the active drug 5-FdU was released exclusively within the target cells [27]. Thus, the aptamer did act as a prodrug as it fulfilled two main prerequisites of a drug delivery system: specific cell targeting and controlled release of the drug triggered by an endogenous stimulus. As this prodrug also could be enzymatically reverse transcribed into DNA, which then served as template for the synthesis of new prodrug molecules, it thus also functioned as its own gene. 

### 2.5. Structural Investigations of IL-6R Aptamers

**Figure 7:**
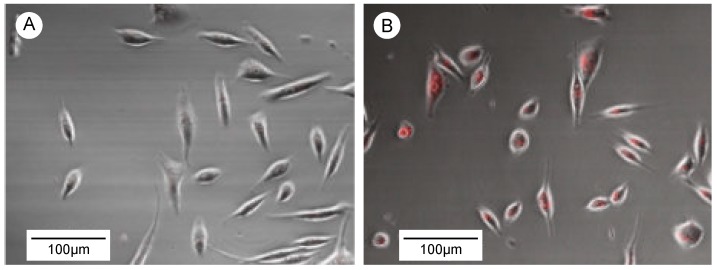
Fluorescently labeled aptamer IDA is internalized by integrin presenting PC-3 cells. Cells were incubated with fluorescently labelled non-specific DNA (**A**) and aptamer IDA (**B**) as described [41]. Scanning microscopic analysis after treating both samples with DNase showed clearly labelled molecules inside cells.

Remarkably, all IL-6R specific aptamers selected in our laboratory—regardless whether consisting of DNA or RNA—at least partly comprise a G-quadruplex structure (Figure 3; [4,5,6,7,40]). One might get the impression that this structural motive is a prerequisite for a nucleic acid aptamer for binding to IL-6R [40]. This is especially striking in case of the SELEX-selected IL-6R specific only 16 nt long DNA aptamer d(GGGT)_4_ whose RNA counterpart r(GGGU)_4_ also behaves very similar with respect to IL-6R binding and inhibition of HIV-1 integrase and HIV-1 infection [7].

Further structural investigations of the different aptamers discussed here included small-angle X-ray scattering (SAXS) analyses, structure probing, electrophoretic mobility shift assays and microscale thermophoresis [6,40]. In all cases the investigated aptamers were shown to form dimers (Figure 3).

## 3. Integrin α6β4 Specific DNA Aptamer IDA—Another Charomer

In another project in our laboratory we selected IDA, a 77 nt long integrin α6β4 specific DNA aptamer [41]. The initial motivation for the selection of IDA was to get a tool at hand to inhibit α6β4 integrin mediated cell-cell-interactions. Especially as this particular interaction can constitute a pivotal step in transendothelial migration during metastasis formation [8,11,42,43]. This aptamer actually binds its target (*K*_d_ about 140 nM) and also blocks the integrin-laminin-interaction but it is also internalized very effectively. Under appropriate conditions 98% of fluorescently labelled aptamer was internalized within 10 min (Figure 7; [41]).

## 4. Conclusions

The nucleic acid charomers described here are targeting two different cell surface transmembrane proteins exhibiting different functions. The initial motivation for selecting these aptamers was to get tools at hand to inhibit the best-known functions of their targets—receiving signals from other cells or mediating unfavorable cellular interactions. As the targeted proteins are not solely presented but also internalized by the producing cells, it was not surprising that the mentioned and quite strongly binding nucleic acid aptamers were concurrently internalized, too. The possibility to fuse different kinds of cargo molecules [25,44] or even larger particles [21,45,46] with these internalized aptamers makes them charomers.

The inhibitory efficiency of the charomers reported here may not yet be very satisfactory but they can be precursors of a potentially very helpful new class of therapeutics and possibly their effect could be enhanced by the combination of several different charomers, covalently linked to each other or just in an appropriate mixture. Also attempts to improve stability and pharmacokinetic of the charomers might increase their utility. One can think about many different possibilities of new selection strategies or chemical modifications [6,47,48]. Lastly, the applicability in the living organism has to be demonstrated. 

And now one final idea. If one imagines how many nucleic acid molecules can be found in the environment (early described by Karl and Bailiff [49]) one easily can imagine that not only a few of them will find their way from the environment into a cell just due to an accidentally sufficient affinity to a surface protein which is internalized. Maybe this is another noteworthy passway for gene exchange across species barriers.
